# β-Hydroxybutyrate Oxidation in Exercise Is Impaired by Low-Carbohydrate and High-Fat Availability

**DOI:** 10.3389/fmed.2021.721673

**Published:** 2021-11-25

**Authors:** David J. Dearlove, David Holdsworth, Tom Kirk, Leanne Hodson, Evelina Charidemou, Eline Kvalheim, Brianna Stubbs, Andrew Beevers, Julian L. Griffin, Rhys Evans, Jeremy Robertson, Kieran Clarke, Pete J. Cox

**Affiliations:** ^1^Department of Physiology, Anatomy and Genetics, University of Oxford, Oxford, United Kingdom; ^2^Oxford Centre for Diabetes, Endocrinology and Metabolism, Oxford NIHR Biomedical Research Centre, University of Oxford, Oxford, United Kingdom; ^3^Department of Biochemistry and Cambridge Systems Biology Centre, University of Cambridge, MRC Human Nutrition Research, Cambridge, United Kingdom; ^4^Department of Chemistry, University of Oxford, Oxford, United Kingdom; ^5^Research and Development Department, Sterling Pharma Solutions Ltd., Cramlington, United Kingdom

**Keywords:** ketone, ketosis, exogenous ketosis, oxidation, exercise

## Abstract

**Purpose:** In this study, we determined ketone oxidation rates in athletes under metabolic conditions of high and low carbohydrate (CHO) and fat availability.

**Methods:** Six healthy male athletes completed 1 h of bicycle ergometer exercise at 75% maximal power (WMax) on three occasions. Prior to exercise, participants consumed 573 mg·kg bw^−1^ of a ketone ester (KE) containing a ^13^C label. To manipulate CHO availability, athletes undertook glycogen depleting exercise followed by isocaloric high-CHO or very-low-CHO diets. To manipulate fat availability, participants were given a continuous infusion of lipid during two visits. Using stable isotope methodology, β-hydroxybutyrate (βHB) oxidation rates were therefore investigated under the following metabolic conditions: (i) high CHO + normal fat (KE+CHO); (ii) high CHO + high fat KE+CHO+FAT); and (iii) low CHO + high fat (KE+FAT).

**Results:** Pre-exercise intramuscular glycogen (IMGLY) was approximately halved in the KE+FAT vs. KE+CHO and KE+CHO+FAT conditions (both *p* < 0.05). Blood free fatty acids (FFA) and intramuscular long-chain acylcarnitines were significantly greater in the KE+FAT vs. other conditions and in the KE+CHO+FAT vs. KE+CHO conditions before exercise. Following ingestion of the ^13^C labeled KE, blood βHB levels increased to ≈4.5 mM before exercise in all conditions. βHB oxidation was modestly greater in the KE+CHO vs. KE+FAT conditions (mean diff. = 0.09 g·min^−1^, *p* = 0.03; *d* = 0.3), tended to be greater in the KE+CHO+FAT vs. KE+FAT conditions (mean diff. = 0.07 g·min^−1^; *p* = 0.1; *d* = 0.3) and were the same in the KE+CHO vs. KE+CHO+FAT conditions (*p* < 0.05; *d* < 0.1). A moderate positive correlation between pre-exercise IMGLY and βHB oxidation rates during exercise was present (*p* = 0.04; *r* = 0.5). Post-exercise intramuscular βHB abundance was markedly elevated in the KE+FAT vs. KE+CHO and KE+CHO+FAT conditions (both, *p* < 0.001; *d* = 2.3).

**Conclusion:** βHB oxidation rates during exercise are modestly impaired by low CHO availability, independent of circulating βHB levels.

## Introduction

Ketogenesis is a vital metabolic adaptation to starvation (1). The hepatic synthesis of ketone bodies [mainly β-hydroxybutyrate (βHB) and acetoacetate (AcAc); herein collectively referred to as ketones] predominately from free fatty acid (FFA) links energy stored in adipose tissue to cerebral metabolism (2) under conditions of reduced carbohydrate (CHO) intake. Indeed, ketones replace glucose as the primary metabolic substrate during prolonged starvation (2). Whilst the teleological purpose of ketogenesis is to provide a supplementary fuel for the brain (1, 2), most oxidative tissues can metabolize ketones (3). Given its substantial contribution to overall body mass, skeletal muscle is the largest site of ketone uptake and oxidation (3), particularly during exercise (4–6) when metabolic rate is markedly elevated (7). Accordingly, there is interest in whether nutritional ketones, arguably most effectively delivered as a ketone ester (KE) (8), could provide a supplementary fuel to power muscular contraction (9). However, the contribution of exogenous ketone oxidation to whole-body energy expenditure appears to be low in exercising humans, with estimates ranging from 0 to 10% (4, 6, 10, 11).

Fuel selection within tissues is controlled by dynamic interactions between substrates (12). For example, the glucose-fatty acid cycle (Randle cycle) describes the inhibition of glucose oxidation by fatty acids (and ketones) and vice versa (12, 13). Indeed, ketones are “out competed” by CHO- and lipid-derived substrates for oxidation in isolated mitochondria (14). It is plausible, therefore, that ketone oxidation rates might be greater in glycogen depleted muscle, where CHO competition for oxidation would theoretically be decreased. However, further consideration is that glycogen is a major source of anaplerotic substrate for the Krebs cycle (15), the common pathway in which acetyl-CoA derived from glycolysis, β-oxidation, and ketolysis is oxidized. Thus, in glycogen-depleted states, such as prolonged vigorous exercise (16) or low CHO intake (17), ketone oxidation might be impaired.

To determine the effect of circulating and intramuscular substrate concentrations on ketone metabolism, the present study measured βHB oxidation rates in athletes during exercise under conditions of low and high CHO and fat availability. In doing so, we sought to determine the optimal metabolic conditions under which ketone metabolism might support muscular contraction.

## Materials and Methods

### Subjects

**Figure 1:**
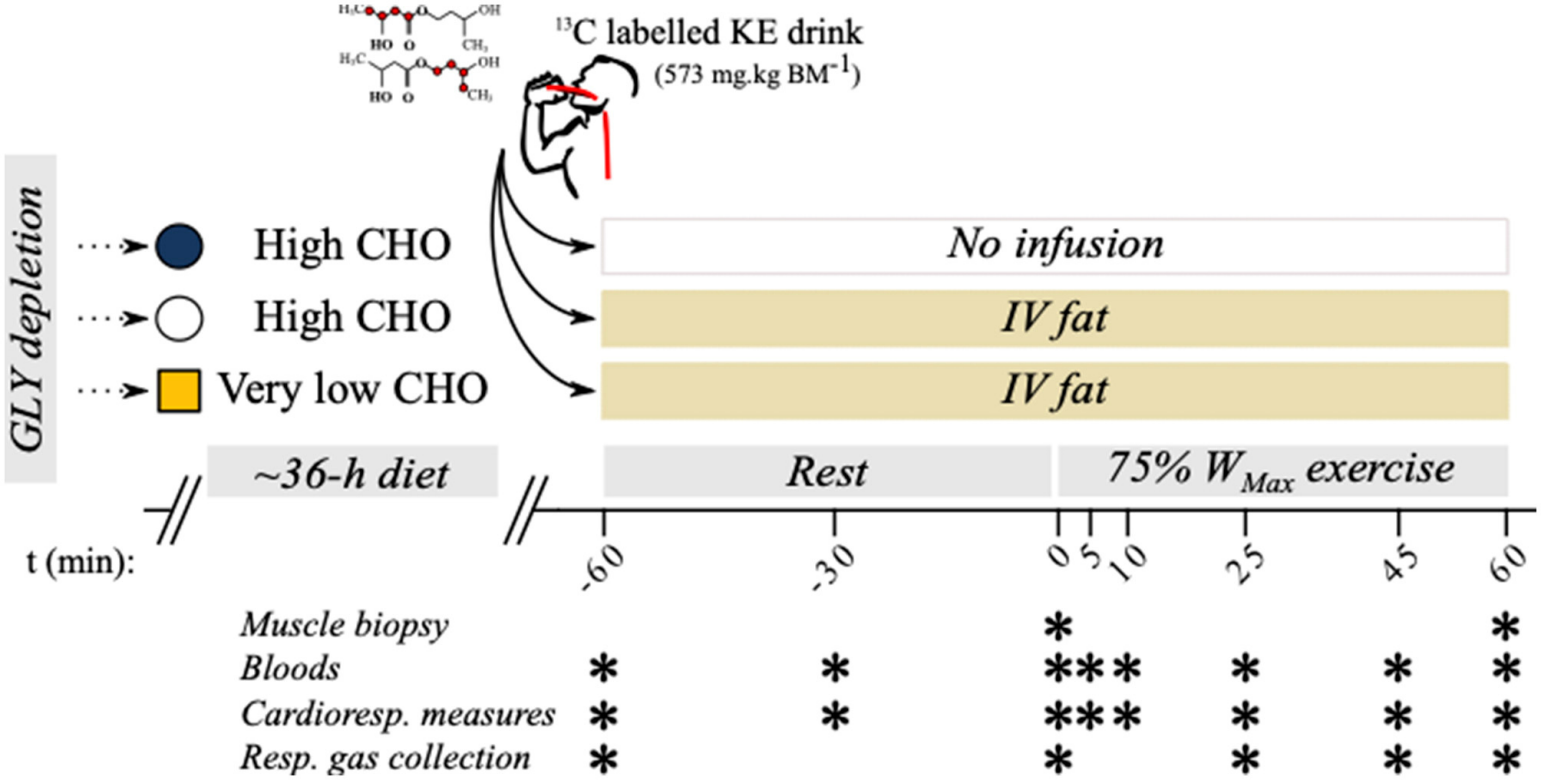
Study protocol overview. Six well-trained athletes were recruited to a 3-way crossover design study where oxidation rates were measured during exercise following manipulations to carbohydrate (CHO) and fat availability.

Six male endurance athletes (Table 1) were recruited to a random-order controlled, cross-over-design study (Figure 1). One participant withdrew consent for muscle biopsies at two visits (KE+CHO+FAT and KE+FAT conditions) and as such, their muscle data was not included in final analyses. Ethical approval for the study was acquired from the Oxfordshire Regional Ethics Committee. All subjects provided written informed consent prior to participation.

**Table 1:** Participant characteristics.

	**Mean ± SD**
Age (yr)	35, 5
Height (cm)	184, 13
Weight (kg)	82, 17
BMI (kg m^2^)	23.8, 2.5
VO_2 Max_ (L min^−1^)	4.8, 0.7
VO_2 Max_ (mL kg BW^−1^)	59.0, 7.6
W_Max_	348, 44

Athletes were recruited by direct invitation or having responded to targeted advertisements. Eligibility criteria were men or women aged from 18 to 40 years; undertaking ≥10 h·wk^−1^ endurance-exercise training for the 3 months prior to enrollment; and being injury and illness free at the time of enrollment. The study exclusion criteria were any previous history of cardiovascular, neuromuscular, endocrine or neurological disease, or any other medical condition requiring long-term medication; smoking of any kind; and pregnancy or current breastfeeding, or female athletes not taking the oral contraceptive pill.

### Baseline Testing

A baseline exercise test was undertaken to determine individual exercise intensities [% of maximal power (*W*_Max_)] for subsequent study visits. Participants arrived at the Department of Physiology, Anatomy and Genetics (DPAG), the University of Oxford having fasted overnight (8 to 10 h fast). The exercise was performed on an electronically-braked cycling ergometer (Ergoselect 100, Ergoline, Germany). Participants adjusted the handlebar position and saddle height according to personal preference. Individual bike geometry was recorded and kept consistent across all subsequent visits. The ergometer was set to the revolutions per min (RPM)-independent mode, meaning resistance was constant regardless of the cadence held. Water was allowed *ad libitum* before exercise. The exercise was commenced at 100 W and increased 25 W every 3 min until volitional fatigue. To ensure exercise was undertaken to maximal capacity, two of the following criteria must have been met:

- upon an increase in exercise intensity, the volume of oxygen consumed (VO_2_) was not elevated by more than 0.2 L·min^−1^;
- heart rate (HR) was within 10 beats per min (BPM) of an age predicted maximum (220-age);
- the respiratory exchange ratio (RER) was >1.1; and
- an inability to maintain a cadence >60 RPM.

Study investigators provided verbal encouragement during exercise. Respiratory gases were measured continuously through indirect calorimetry. Maximum oxygen uptake (VO_2*Max*_) was defined as the highest 20 s average for VO_2_. Maximal power In review (W_*Max*_) was calculated according to the formula (18):


$$W_{\text{Max}}=W_{Step\;N-1}+\left(time\;elapsed/180\;sec\cdot 25W\right)$$

### Study Overview

β-hydroxybutyrate oxidation rates were measured during exercise following manipulation of dietary CHO and circulating FFA levels. The three experimental conditions (Figure 1) studied were as follows:

- KE + high-CHO diet + normal fat (KE+CHO);
- KE + high-CHO diet + high fat (KE+CHO+FAT); and
- ketone ester (KE) + low-CHO diet + high fat (KE+FAT).

Participants undertook these conditions in a random order, which was determined following enrollment.

### CHO Availability Manipulations

Thirty-six hours prior to each study visit, participants undertook a glycogen depleting exercise protocol on the same bicycle ergometer (Ergoselect 100, Ergoline, Germany). This consisted of alternating 2 min “work” and “recovery” intervals commencing at 90 and 50% W_*Max*_, respectively. When the participant could no longer hold 90% W_*Max*_ for 2 min (volitional fatigue or inability to hold a cadence ≥60 RPM), the work intensity was reduced by 10%. This continued until participants could no longer maintain 60% W_*Max*_ during work intervals. Participants were instructed to hold a cadence of 80 to 100 RPM, and the bicycle ergometer was set to the RPM-independent mode. This protocol reliably depletes intramuscular glycogen (IMGLY) stores (19).

For the high-CHO availability conditions (KE+CHO and KE+CHO+FAT), participants then adopted a high-CHO diet (target of 7 g·kg bw^−1^) lasting until the evening before the study visits (refer to Supplementary Information 1 for an example diet diary). Athletes were familiar with the practice of pre-race CHO loading. This level of CHO intake is in-line with current recommendations to support IMGLY restoration during exercise training (6 to 12 g·kg bw^−1^) (20). For the low-CHO availability visit (condition: KE+FAT), participants adopted a strict very-low-CHO diet (target of ≤ 5% total energy derived from CHO), with the goal of ensuring that intramuscular glycogen (IMGLY) was not fully replenished (21). For all conditions, participants were supplied with food and drink items and kept a diet diary.

### Study Visits

Participants attended DPAG having fasted overnight (8 to 10 h fast). A 22-gauge catheter (BD Venflon™ Pro Safety; BD, UK) was inserted into an antecubital vein of the arm. A three-way tap (BD Connecta™ Plus Stopcock; BD) was attached, and a baseline blood sample was collected into an ethylenediaminetetraacetic acid (EDTA) tube (*t* = -60 min). Blood samples were stored in an ice water bath until the completion of the visit. Resting respiratory gases and HR were measured. Respiratory gas samples were collected using a Douglas bag. A second 22-gauge catheter was inserted into an antecubital vein on the contralateral arm for the continuous infusion of 20% Intralipid® (20% soybean oil, 1.2% egg yolk phospholipids, and 2.25% glycerin and water; Baxter Healthcare, US) at 50 mL·h^−1^ for the duration of the visit. Participants then consumed a drink containing 573 mg·kg bw^−1^ of βHB monoester (d-βHB-*R*-1,3-butanediol monoester; TΔS, UK) diluted in water. A weight adjusted volume of a bespoke ^13^C KE tracer was added to the drinks, providing a relative enrichment of ≈50%. The methods used to synthesize the ^13^C tracer have been described previously (6). Participants were then prepared for a biopsy of the vastus lateralis muscle using an aseptic technique previously described (22). Samples were attained from separate incisions spaced ≈5 cm apart before and immediately after exercise. Tissue was frozen immediately in liquid nitrogen and subsequently stored at −80°C. Further resting (post-drink) blood samples and cardiorespiratory measures were taken at *t* = –30 and 0 min. A resting respiratory gas sample was collected at *t* = 0 min. Participants then undertook 60 min of cycling exercise at 75% W_*Max*_. Blood samples, cardiorespiratory measures, and ratings of perceived exertion (RPE) were taken at *t* = 5, 10, 25, 45, and 60 min during exercise. Respiratory gas samples were collected *via* a Douglas bag during exercise at *t* = 25, 45, and 60 min.

### Blood Sampling and Analysis

β-hydroxybutyrate concentration was analyzed immediately from venous blood samples (Freestyle Optium Neo, Abbott Laboratories, USA). Blood samples were centrifuged (10 min at 3,600 RPM, 4°C). The serum component was transferred to Eppendorf tubes, which were stored at −25°C until later analysis. Samples were analyzed for glucose, lactate, and FFA using a semi-automated bench top analyser (Pentra C400, Horiba Medical, France).

### Cardiorespiratory Measurements

Participants wore a snug fitting face mask (Hans Rudolph, USA) attached to a calibrated indirect calorimeter (Metalyzer 3BR2, Cortex Biophysik, Germany) for the measurement of respiratory gas compositions and ventilatory flow. Values were displayed in real time using Metasoft® (v7.9.1, Cortex Biophysik, Germany). Subjects wore a Polar HR monitor (T31, Polar Electro, Finland) that communicated with the Metalyzer *via* a receiver cable attached to the ergometer. Cardiorespiratory data was exported in Excel® (Microsoft Corporation, USA) for further analysis. Clearly, aberrant values were omitted, and a minimum 30 s average was taken for each measurement point.

### Respiratory Gas Sampling

Immediately following pulmonary gas measurements at *t* = –60, 0, 25, 45, and 60 min, participants breathed into a mouthpiece containing a one-way valve that was connected to a 50 L Douglas bag. Duplicate respiratory gas samples were aspirated *via* a sealed port into 12 ml Exetainer^TM^ vials (Labco, UK). Vials were subsequently stored at room temperature.

### Gas Chromatography-Mass Spectrometry

The ^13^C enrichment in drinks was determined by gas chromatography-mass spectrometry after derivatisation with a trimethylsilyl group (23). The tracer to tracee ratio (TTR) (^13^CO_2_ /^12^CO_2_) in respired gases was determined using previously published methods (24).

### Calculation of βHB Oxidation

β-hydroxybutyrate oxidation rate (g·min^−1^) was estimated from the TTR using the following equation.


$$\begin{array}{l} VCO_{2}((\text{TTR}CO_{2\text{expired}}-\text{TTR}CO_{2\text{pre-drink}})/\\ \left({\text{TTR}}_{\text{labelled drink}}-{\text{TTR}}_{\text{unlabelled drink}}\;))(1/\kappa \right) \end{array}$$

The molar mass of βHB was assumed to be 104.1 g·mol^−1^ and κ = the volume of^13^CO_2_ produced from the complete oxidation of 1 g of βHB (0.86 L).

### Determination of IMGLY

Glycogen extraction from muscle samples was performed according to previously published methods (25). Briefly, muscle samples (≈15 mg) were freeze dried within fenestrated Eppendorf tubes for 72 h. Dry weights were recorded for each sample, and samples were transferred to homogenization vials (product code: CK28, Bertin Instruments, UK) containing 500 μl of 1 M HCl. Samples were homogenized in a Precellys tissue homogenizer (Bertin Instruments, UK) and placed in an 80°C water bath for 2 h with occasional vortexing. Once cooled to room temperature, samples were neutralized through the addition of 250 μl Tris/KOH saturated with KCl. Samples were then centrifuged for 15 min (4°C, 14,000 g), and the supernatant was removed. Finally, samples were assayed for glucose using the hexokinase method on a semi-automated benchtop analyser (Pentra C400, Horiba Medical, France). Glycogen measurements were determined in a single muscle biopsy sample for each participant.

### Muscle Metabolite Analysis

Glycolytic and Krebs cycle metabolites, carnitine species, and β-hydroxybutyric acid were measured in pre- and post-exercise muscle biopsy samples by liquid chromatography-mass spectrometry. Refer to Supplementary Information 2 for a detailed description of the methods employed.

### Statistics

Change in blood βHB concentration was used as a surrogate measure of βHB oxidation for the purpose of *a priori* power calculations. We have previously established that consuming 573 mg·kg bw^−1^ KE before exercise causes blood βHB levels to reach (mean ± SD) 3.42 mM ± 0.21 mM after 60 min of exercise at 75% WMax (22). We powered our study to detect a 10% increase in blood βHB in the glycogen-depleted vs. glycogen-repleted state. This equated to an effect size f = ≈0.7. Using G*Power (26), with an alpha probability of 0.05 and power of 0.8, we required *n* = 6 participants within a 3 group, repeated measures, within factors design study. The present work was conducted before our laboratory's published investigation into the effects of blood βHB concentration and exercise intensity on βHB oxidation rates (6) and as such, we did not have this data available for power calculations.

Significance was determined as *p* < 0.05. Unless otherwise stated, data are presented as mean differences with 95% CI and Cohen's d effect sizes within the text and mean ± SD within figures. All data were analyzed in Prism version 9.0.2 (GraphPad Software, USA). Where data did not meet parametric test assumptions, log transformations were applied (all analyses were performed on the transformed data, but non-transformed data are presented in Figures and Results) or appropriate non-parametric tests were used. A one-way repeated measures ANOVA was performed where a single independent variable containing multiple levels was present. A two-way repeated measures ANOVA was used when multiple independent variables were present. Mixed effects models were used to analyse repeated measures data with missing data points. Significant ANOVA effects are reported within the text and/or figures. Appropriate *post-hoc* comparisons were used to investigate main and simple effects. Correlations were investigated through Pearson correlation coefficients.

## Results

### Substrate Manipulations

Time to exhaustion during glycogen depleting exercise, performed 36 h before study visits, was comparable between conditions (KE+CHO = (mean ± SD) 116 ± 17 min, KE+CHO+FAT = 105 ± 15 min, and KE+FAT = 117 min ± 15 min). Participant-reported pre-visit (≈36 h) dietary macronutrient compositions were comparable between the high CHO availability conditions (KE+CHO and KE+CHO+FAT), with CHO representing ≈65% of total energy intake (Supplementary Information 3). Conversely, the contribution of CHO to overall energy intake was just ≈3% in the KE+FAT condition. Total energy intake was comparable between all conditions.

**Figure 2:**
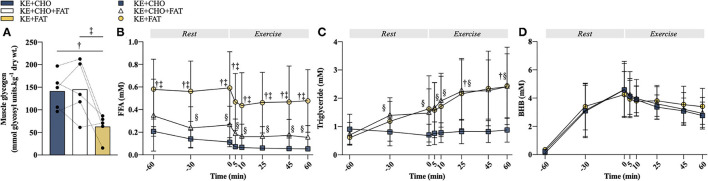
Substrate manipulations. **(A)** Pre-exercise intramuscular glycogen (IMGLY). **(B)** Plasma free fatty acid (FFA) concentration at rest and during exercise. **(C)** Plasma triglyceride concentration at rest and during exercise. **(D)** Blood βHB concentration at rest and during exercise. Significant *post-hoc* comparisons: † = ketone ester (KE)+CHO vs. KE+FAT; ‡ = KE+CHO+FAT vs. KE+FAT; § = KE+CHO vs. KE+CHO+FAT. Values = mean ± SD.

Dietary manipulations caused deviations in pre-exercise IMGLY content (Figure 2A; ANOVA, *p* = 0.006). Pre-exercise IMGLY was approximately halved in the KE+FAT vs. KE+CHO (mean diff. = 78.6 mmol glycosyl units·kg dry wt^−1^; 95% CI = 19.8 mmol glycosyl units·kg dry wt^−1^ to 137.4 mmol glycosyl units·kg dry wt^−1^; *d* = 2.2; *p* = 0.04) and KE+CHO+FAT (mean diff. = 82.4 mmol glycosyl units·kg dry wt^−1^; 95% CI = 23.6 mmol glycosyl units·kg dry wt^−1^ to 141.2 mmol glycosyl units·kg dry wt^−1^; *d* = 1.7; *p* = 0.01).

The very-low-CHO diet also raised circulating FFA (Figure 2B; ANOVA: Time*Condition Interaction, *p* < 0.001), with plasma FFA concentration being greater at *t* = –60 min (fasted) in the KE+FAT vs. KE+CHO (mean diff. = 0.42 mM; 95% CI = 0.26 to 0.57 mM; *d*=2.0; *p* < 0.001) and KE+CHO+FAT (mean diff. = 0.30 mM; 95% CI = 0.15 to 0.45 mM; *d* = 0.8; *p* < 0.001) conditions. Following commencement of the intravenous lipid infusion in the KE+CHO+FAT and KE+FAT conditions, plasma FFA levels were significantly different between all conditions at rest and throughout exercise. The intravenous lipid infusion also raised circulating triglyceride levels (ANOVA: Time*Condition Interaction, *p* 0.01), which were the same in all conditions pre-infusion (*t* = 60 min), but significantly greater in the KE+CHO+FAT vs. KE+CHO condition at all measurements post-infusion, and significantly greater in the KE+FAT vs. KE+CHO condition at *t* = 25 and 60 min during exercise (Figure 2C).

Consumption of 573 mg·kg bw^−1^ KE caused blood βHB to rise similarly in all conditions, peaking at ≈4.5 mM pre-exercise (Figure 2D). Blood βHB concentration was comparable between conditions throughout the exercise. Taken together, study interventions created three distinct metabolic conditions in which to investigate βHB oxidation.

### βHB Oxidation

**Figure 3:**
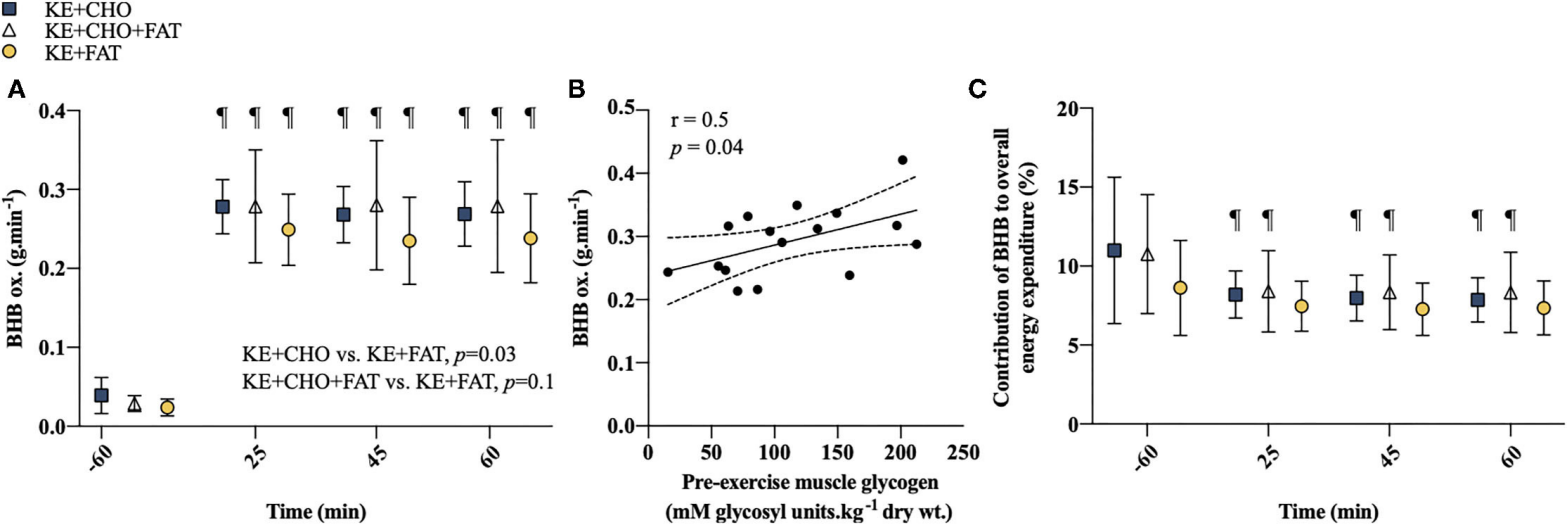
βHB oxidation. **(A)** βHB oxidation rates at rest and during exercise. **(B)** Association between pre-exercise IMGLY and mean βHB oxidation rates during exercise. Limits represent 95% CI. **(C)** Contribution of βHB to overall energy expenditure at rest and during exercise. Significant *post-hoc* comparisons: ¶ = within group, vs. *t* = −60 min (fasted). Values = mean ± SD.

A significant Condition effect was present for βHB oxidation rates (*p* = 0.03; Figure 3A), which were greater in the KE+CHO vs. KE+FAT conditions (mean diff. = 0.09 g·min^−1^; 95% CI = 0.01 to 0.17 g·min^−1^; *d* = 0.3; *p* = 0.03) and tended to be greater in the KE+CHO+FAT vs. KE+FAT conditions (mean diff. = 0.07 g·min^−1^; 95% CI = –0.01 to 0.15 g·min^−1^; *d* = 0.3; *p* = 0.1). However, exploratory *post-hoc* analyses suggested that βHB oxidation rates were greater in the KE+CHO+FAT vs. KE+FAT condition at all time points during exercise, with similar effect sizes to the KE+CHO vs. KE+FAT comparisons (refer to Supplementary Information 4). There was no difference in βHB oxidation rates between high-CHO availability conditions with or without the lipid infusion (mean diff. = 0.02 g·min^−1^; 95% CI = –0.06 to 0.11 g·min^−1^; *d* < 0.1; *p*=0.7). A main effect of time was also present (*p* < 0.001), with βHB oxidation rates being markedly increased during exercise vs. rest in all conditions (all *p* < 0.001). During exercise, βHB oxidation rates remained stable in all conditions. There was a moderate positive correlation between pre-exercise IMGLY and mean βHB oxidation rates during exercise (*r* = 0.5; *p* = 0.04; Figure 3B). A main effect of Time was present for the contribution of βHB to overall energy expenditure (*p* = 0.04; Figure 3C). In the KE+CHO and KE+CHO+FAT conditions, the contribution of βHB to overall energy expenditure fell from rest to all exercise measurements (all *p* < 0.001). However, this effect was not present in the KE+FAT condition.

### Cardiorespiratory and Exertion Measurements

**Figure 4:**
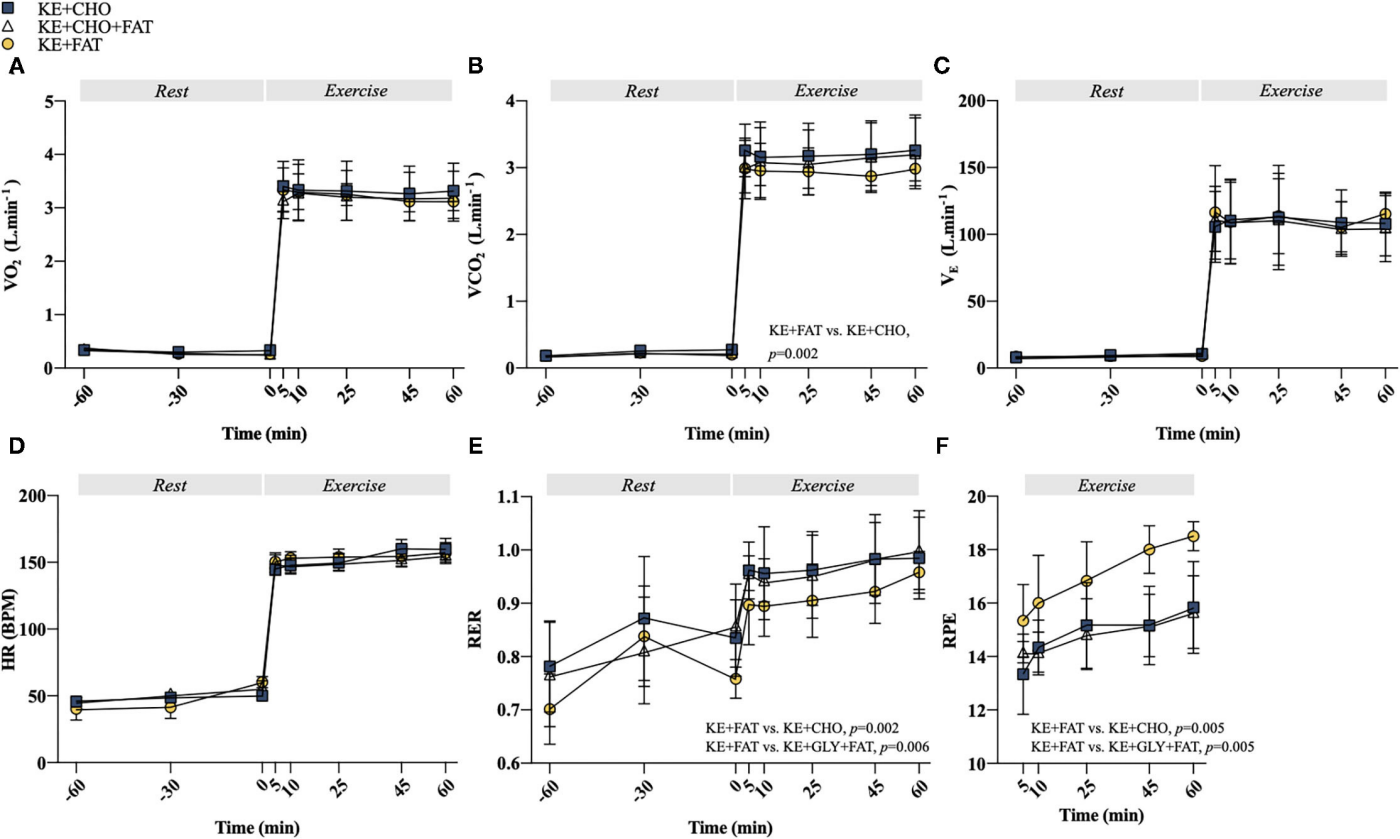
Cardiorespiratory and exertion measurements. **(A)** Volume of oxygen consumed (VO_2_) at rest and during exercise. **(B)** Volume of carbon dioxide expelled (VCO_2_) at rest and during exercise. **(C)** Ventilatory exchange (V_E_) at rest and during exercise. **(D)** Heart rate (HR) at rest and during exercise. **(E)** Respiratory exchange ratio (RER) at rest and during exercise. **(F)** Ratings of perceived exertion (RPE) at rest and during exercise. Values = mean ± SD.

Exercise stimulated an increase in VO_2_, ventilatory exchange (VE), and heart rate (HR); however, no differences between conditions were observed at any time point (Figures 4A,C,D). A Condition effect was present for the volume of carbon dioxide expelled (VCO_2_) (*p* = 0.003; Figure 4B), which was greater in the KE+CHO vs. KE+FAT conditions (mean diff. = 0.05 L·min^−1^; 95% CI = 0.02 to 0.07 L·min^−1^; *d* = 0.1; *p* = 0.002). There was also a Condition effect for RER (*p* = 0.002; Figure 4E), which was lower (indicating greater fat oxidation) in the KE+FAT conditions (mean diff. = 0.06; 95% CI = 0.02 to 0.09; *d* = 0.6; *p* = 0.002) and KE+CHO+FAT conditions (mean diff. = 0.05; 95% CI = 0.02 to 0.08; *d* = 0.5; *p* = 0.006). Perceived exertion during exercise was greater following the adoption of the very-low-CHO diet (Figure 4F; ANOVA: Condition, *p*=0.009), being elevated in the KE+FAT vs. KE+CHO (mean diff. = 2.2; 95% CI = 0.7 to 3.6; *d* = 1.3; *p* = 0.005) and KE+CHO+FAT conditions (mean diff. = 2.1; 95% CI = 0.7 to 3.6; *d* = 1.5; *p* = 0.005).

### Blood Metabolites

**Figure 5:**
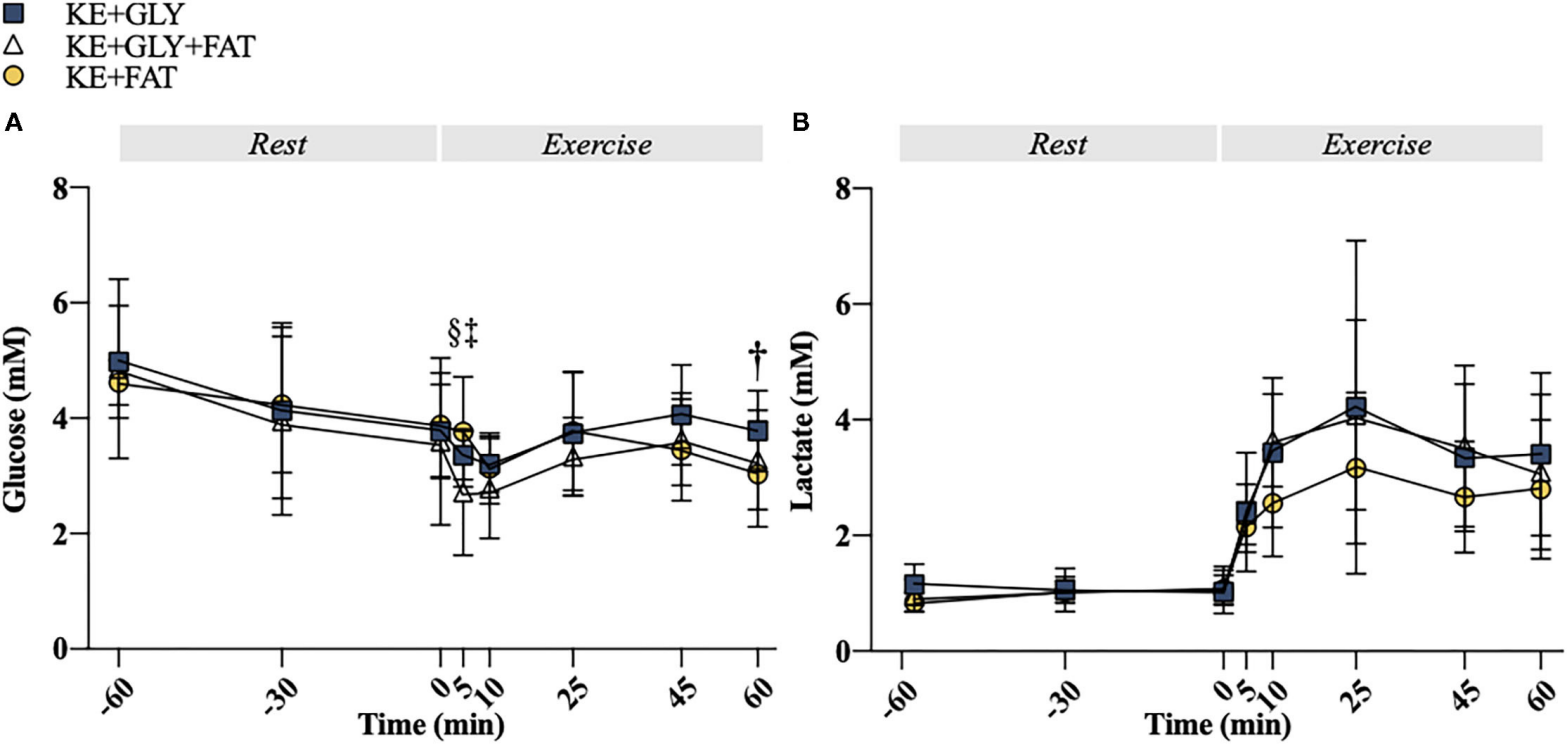
Plasma metabolites. **(A)** Plasma glucose at rest and during exercise. **(B)** Plasma lactate at rest and during exercise. Significant *post-hoc* comparisons: † = KE+CHO vs. KE+FAT; ‡ = KE+CHO+FAT vs. KE+FAT; § = KE+CHO vs. KE+CHO+FAT. Values = mean ± SD.

At *t* = 5 min, blood glucose was lower in the KE+CHO+FAT vs. KE+CHO (mean diff. = 0.64; 95% CI = 0.02 to 1.25; *d* = 0.8; *p*=0.04) and KE+FAT (mean diff. = 1.04; 95% CI = 0.43 to 1.66; *d* = 1.0; *p* < 0.001) conditions. Blood glucose was also lower in the KE+FAT vs. KE+CHO conditions at *t* = 60 min (mean diff. = 0.74; 95% CI = 0.13 to 1.37; *d* = 0.3; *p* = 0.01; ANOVA, Time*Condition Interaction, *p* = 0.04; Figure 5A). No between group differences were observed for blood lactate (Figure 5B).

### Intramuscular βHB

**Figure 6:**
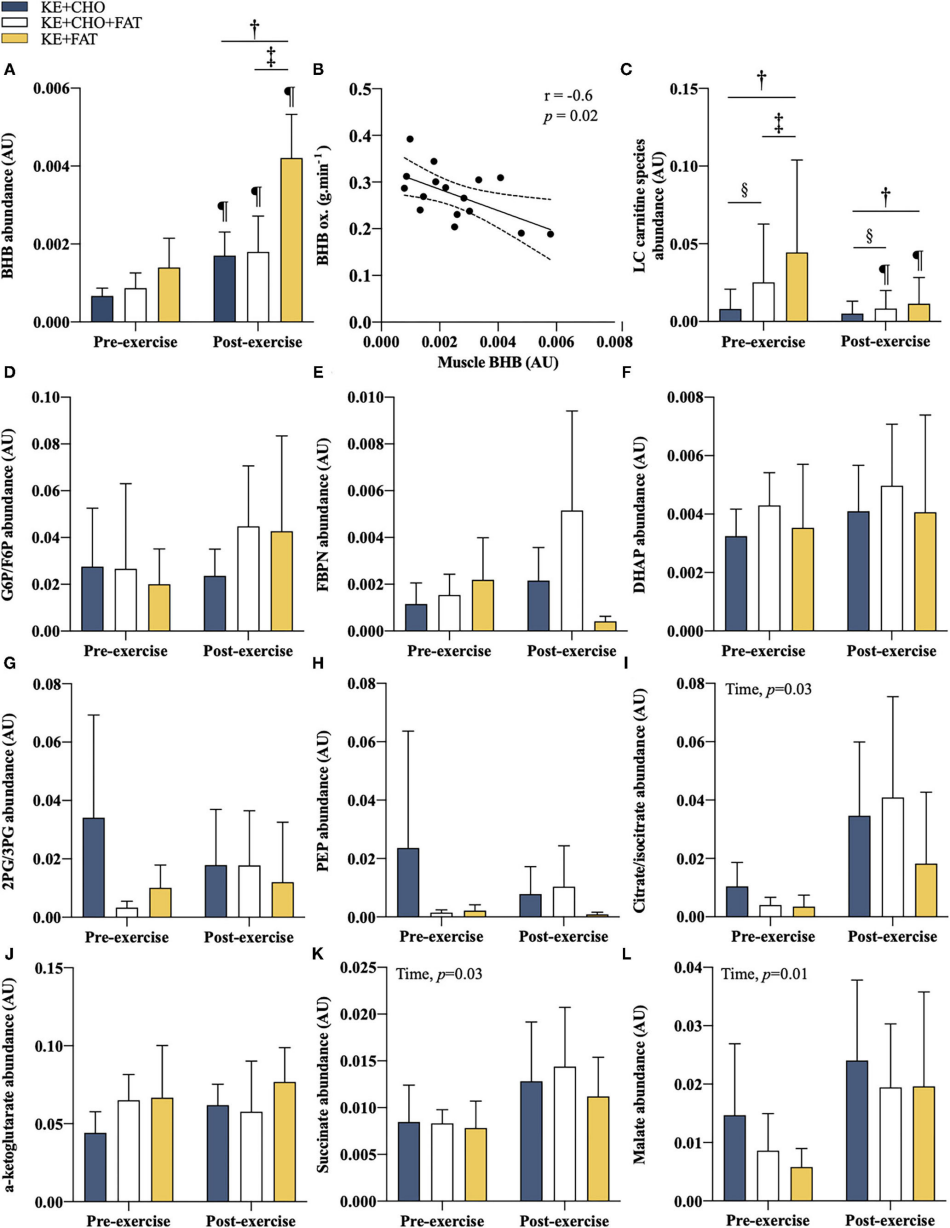
Intramuscular metabolites. **(A)** βHB pre- and post-exercise. **(B)** Association between post-exercise intramuscular βHB and βHB oxidation rate at *t* = 60 min. Limits represent 95% CI. **(C)** Long-chain acylcarnitines (C12 to C18) abundance pre- and post-exercise. **(D)** glucose 6 phosphate (G6P)/fructose 6-phosphate (F6P) pre- and post-exercise. **(E)** fructose 1,6-bisphosphate (FBPN) pre- and post-exercise. **(F)** dihydroxyacetone phosphate (DHAP) pre- and post-exercise. **(G)** 2-Phosphoglyceric acid (2PG)/3-Phosphoglyceric acid (3PG) pre- and post-exercise. **(H)** phosphoenolpyruvic acid (PEP) pre- and post-exercise. **(I)** Citrate/isocitrate pre- and post-exercise. **(J)** α-ketoglutarate pre- and post-exercise. **(K)** Succinate pre- and post-exercise. **(L)** Malate pre- and post-exercise. Significant *post-hoc* comparisons: † = KE+CHO vs. KE+FAT; ‡ = KE+CHO+FAT vs. KE+FAT; § = KE+CHO vs. KE+CHO+FAT; ¶ = Pre- vs. post-exercise. Values = mean ± SD.

Intramuscular βHB abundance was comparable in all conditions in pre-exercise muscle samples (Figure 6A). Exercise stimulated an increase in intramuscular βHB in the KE+FAT condition only (mean diff. = 0.003 AU; 95% CI = 0.001 to 0.004 AU; *d* = 2.3; *p* < 0.001; ANOVA, Condition*Time Interaction, *p* = 0.03). Post-exercise muscle βHB was markedly elevated in the KE+FAT vs. KE+CHO and KE+CHO+FAT conditions (mean diff. = 0.002; 95% CI = 0.001 to 0.004 AU; *d* = 2.3; *p* < 0.001). There was a moderate negative relationship between intramuscular βHB abundance and βHB oxidation rates (*r* = –0.6, *p* = 0.02; Figure 6B).

### Intramuscular Acylcarnitines, Glycolytic Intermediates, and Krebs Cycle Intermediates

We sought to investigate whether changes in targeted intramuscular metabolic pathways might explain observed differences in βHB oxidation rates. Baseline and post-exercise long-chain acylcarnitine (C12 to C18) abundance was significantly greater in the KE+CHO+FAT and KE+FAT vs. KE+CHO conditions (ANOVA: Condition, *p* < 0.001; Time*Condition Interaction, *p* < 0.001; Figure 6C and Supplementary Information 5 for individual carnitine species comparisons). Notably, their abundance was also greater in the KE+CHO+FAT vs. KE+FAT condition at baseline. Exercise caused a significant decrease in long-chain acylcarnitines in the KE+CHO+FAT and KE+FAT conditions only.

Intramuscular glycolytic intermediates were the same in all conditions (Figures 6D–H). A main ANOVA effect of time was present for citrate, succinate, and malate (all *p* < 0.05; Figures 6I–L), although no within group pre- vs. post-exercise differences were present. No between group differences were observed for measured Krebs cycle intermediates.

## Discussion

The effect of CHO and fat availability on human ketone oxidation was unknown. In the present study, we demonstrated that βHB oxidation in exercising humans is modestly decreased by reduced CHO availability, independent of circulating βHB concentrations.

β-hydroxybutyrate oxidation, measured through the recovery of a ^13^C label in respiratory gases, was impaired in the KE+FAT (low CHO, high fat availability) vs. KE+CHO (high CHO, normal fat availability) conditions. This was supported by an approximately 2-fold increase in post-exercise intramuscular βHB in the KE+FAT vs. KE+CHO conditions. Given that endogenous βHB production is inhibited during exogenously-induced ketosis (27), we suggest this represents a “bottleneck” for ketone oxidation, rather than an increased supply of endogenous βHB from ketogenesis in the KE+FAT condition. The decrease in βHB oxidation occurred independently of circulating βHB levels, which were comparable between conditions pre-exercise (post-drink) and throughout exercise, meaning saturation kinetics were not responsible. Given that oxidation rates were low, only a small amount of βHB was “spared” in KE+FAT condition. Over 60 min of exercise, the total additional βHB oxidized in the KE+CHO vs. KE+FAT conditions was just ≈1.9 g or ≈9 Kcal. For context, total energy expenditure during exercise was estimated to be 991 Kcal and 945 Kcal in the KE+CHO and KE+FAT conditions, respectively. It is unlikely that this degree of ketone “sparing” would meaningfully affect skeletal muscle metabolism. As such, this finding has limited applicability toward the use of exogenous ketones to enhance human physical capacity (9).

To unpick whether low CHO or high fat availability modulated the observed decrease in βHB oxidation rates in the KE+FAT vs. KE+CHO conditions, we included a third experimental condition: KE+CHO+FAT (ketosis with high CHO and fat availability). We found that βHB oxidation was comparable between KE+CHO and KE+CHO+FAT conditions, suggesting that circulating fat concentrations do not influence βHB oxidation rates and by extension, implicating CHO availability as an important mechanism. In support of this, we observed a moderate positive relationship between pre-exercise IMGLY and mean βHB oxidation rates during exercise. Glycogen is a major source of anaplerotic substrate for the Krebs cycle (15). Glucose 6-phosphate (G6P), *via* pyruvate and pyruvate carboxylase, may form oxaloacetate. Oxaloacetate is required for the entry of acetyl-CoA–derived from glycolysis, β-oxidation, and ketolysis, into the Krebs cycle. It is plausible, therefore, that a reduction in muscle glycogen content may impair muscle ketone oxidation rates due to a decrease in oxaloacetate and subsequent slowing of Krebs cycle flux. However, no differences in measured Krebs cycle intermediates (citrate/isocitrate, malate, succinate, and α-ketoglutyrate) were observed between conditions (unfortunately, we were unable to detect oxaloacetate abundance, which would have provided greater insight into the validity of this hypothesis). Furthermore, if a reduction in IMGLY availability was impairing Krebs cycle flux and ketone oxidation, by the same mechanism, fat oxidation would presumably have been decreased (both substrates being reliant on oxidative respiration). Yet, fat oxidation was markedly increased in the KE+FAT condition, as indicated by a lower RER and a significant decrease in post-exercise intramuscular acylcarnitines. Finally, if ketone metabolism were closely associated with IMGLY availability, we might expect βHB oxidation rates to progressively decline during 1 h exercise at 75% W_*Max*_; an intensity at which muscle glycogen is a major contributor to energy expenditure (28). However, βHB oxidation rates were stable throughout the exercise (*t* = 25, 45, and 60 min). In sum, whilst we have demonstrated that βHB oxidation is modestly impaired by low CHO availability, we cannot attribute this to decreased IMGLY content *per se*.

Indeed, two observations prevent us from concluding that βHB oxidation rates were independent of elevated fat. First, βHB oxidation rates only tended to be different between KE+CHO+FAT and KE+FAT conditions (both high fat conditions with either high CHO or low CHO availability, respectively). Notably, though, post-exercise intramuscular βHB was approximately 2-fold greater in the KE+FAT vs. KE+CHO+FAT condition, suggesting increased βHB clearance during exercise following a high- vs. low-CHO diet. We cannot discount, therefore, that the statistical similarity in βHB oxidation rates between KE+CHO+FAT and KE+FAT conditions represents a type II error, particularly given the relatively small sample size studied. This is supported by exploratory *post-hoc* analyses (Supplementary Information 3), which indicated that βHB oxidation was greater in the KE+CHO+FAT vs. KE+FAT condition at all time points during exercise with similar effect sizes to the KE+CHO vs. KE+FAT comparisons. Second, blood FFAs and intramuscular fat (determined by acylcarnitine abundance) were significantly greater in the KE+FAT vs. KE+CHO+FAT condition. It is plausible that had circulating and intramuscular fat been similarly elevated in the KE+CHO+FAT condition, we would have observed a similar attenuation of βHB oxidation rates.

The overall contribution of βHB to oxygen consumption was ≈8.0%, ≈8.4%, and ≈7.4% in the KE+CHO, KE+GLAY+FAT (IMGLY replete conditions), and KE+FAT conditions, respectively. This is consistent with previous estimations of between 0 and 10% (4, 6, 10, 11). Notably, using the same labeled KE, we reported a maximum contribution of βHB to the oxygen consumption of ≈4.5% (6). Inter-individual differences in ketone oxidation rates appear to be high (6), although the factors underlying this have not been fully elucidated. An important modulating factor of βHB oxidation rates may be an individual's training status (that is, trained vs. sedentary) (29). However, both studies recruited similarly well-trained athletes. Drink ^13^C enrichment was approximately doubled in the present study. Yet given that βHB oxidation rates were calculated using TTR from study drinks and respiratory gas samples (see Methods for the calculations), this should not affect estimates (that is, greater drink enrichment should be matched by greater incorporation of the ^13^C label in respiratory gas samples). Samples from this study were measured by gas chromatography-mass spectrometry separately from our previous work and thus we cannot discount measurement/instrument error as a factor. However, all respiratory gas and drink samples from the present study were analyzed as a single batch, meaning any measurement/instrument error is unlikely to effect relative changes in oxidation rates reported here.

Notwithstanding the higher oxidation rates reported here vs. our previous work (6) relative to those reported for CHO and fat (28), the contribution of βHB oxidation to overall energy expenditure was low in all conditions. Thus, the present work provides further support to the prevailing hypothesis that any ergogenic effect of exogenous ketones is derived from their metabolic signaling actions, rather than by providing an additional energetic substrate for exercising muscle (9).

## Limitations and Future Directions

Pre-exercise muscle glycogen levels were 3- to 5-fold lower than expected (30). Indeed, in all conditions (both high and low CHO availability), IMGLY content would be considered low (<200 mmol·kg·dw^−1^) (30). Yet, participants reported consuming ≈11 g CHO·kg bw^−1^, which is in line with the gold-standard recommendations for glycogen restoration during exercise training (20). Thus, it appears likely that muscle glycogen levels were underestimated across all conditions. However, the accuracy of observed ≈70 to ≈80 mmol glycosyl units·kg dry wt^−1^ differences between high- and low-CHO availability conditions (for which this study was primarily concerned) are supported by the following: (i) self-reported pre-visit dietary CHO content being just ≈3% in the KE+FAT condition ≈1 g CHO·kg bw^−1^; (ii) markedly elevated baseline blood FFA and intramuscular acylcarnitine abundance in the KE+FAT condition; (iii) decreased RER in the KE+FAT vs. high CHO availability conditions during exercise at *t* = 10 and 45 min, and; (iv) increased perceived effort during exercise in the KE+FAT condition [low muscle glycogen content being associated with increased exercise fatigue (30)].

Work by Petrick et al. (14) demonstrated that ketones may support mitochondrial respiration, but their contribution is blunted by the addition of CHO- and lipid-derived substrates. The present work was lacking a glycogen depleted condition with low fat availability, which would have helped elucidate whether Petrick and colleagues', *in vitro* findings may be extrapolated to exercising humans and provided further insight into the mechanisms regulating ketone oxidation. However, the cost of the bespoke ^13^C βHB tracer meant that we were prohibited from recruiting a larger sample needed to power a fourth experimental condition. Indeed, the relatively small sample size also means the study is likely underpowered to detect changes in secondary outcome measures, most notably intramuscular metabolite measurements, which had high variation. As such, these measures are susceptible to type II error and should be considered exploratory.

## Conclusion

βHB oxidation rates were modestly impaired by low CHO availability, independent of blood βHB concentration.

## Data Availability Statement

The original contributions presented in the study are included in the article/Supplementary Material, further inquiries can be directed to the corresponding author/s.

## Ethics Statement

The studies involving human participants were reviewed and approved by East of England - Cambridgeshire and Hertfordshire Research Ethics Committee (14/EE/0063). The patients/participants provided their written informed consent to participate in this study.

## Author Contributions

PC and KC: study design. PC, DH, TK, DD, EK, AB, RE, and JR: conducting studies. PC, DD, DH, LH, TK, EC, EK, BS, AB, and JG: analysis. DD, PC, DH, and RE: manuscript preparation. All: manuscript editing.

## Conflict of Interest

KC is the director of TΔS Ltd., a spin-out company of the University of Oxford, established to develop and commercialise products based on the BHB monoester used in this study. DD is a current employee of TΔS Ltd and TK and BS are former employees. AB was employed by the company Sterling Pharma Solutions Ltd. The remaining authors declare that the research was conducted in the absence of any commercial or financial relationships that could be construed as a potential conflict of interest.

## Publisher's Note

All claims expressed in this article are solely those of the authors and do not necessarily represent those of their affiliated organizations, or those of the publisher, the editors and the reviewers. Any product that may be evaluated in this article, or claim that may be made by its manufacturer, is not guaranteed or endorsed by the publisher.

## Abbreviations

2PG: 2-Phosphoglyceric acid
3PG: 3-Phosphoglyceric acid
AcAc: acetoacetate
βHB: V-hydroxybutyrate
BPM: beats per min
CHO: carbohydrate
DHAP: dihydroxyacetone phosphate
DPAG: Department of Physiology, Anatomy and Genetics
EDTA: ethylenediaminetetraacetic acid
F6P: fructose 6-phosphate
FBPN: fructose 1,6-bisphosphate
FFA: free fatty acid
G6P: glucose 6 phosphate
HR: heart rate
IMGLY: intramuscular glycogen
KE: ketone ester
PEP: phosphoenolpyruvic acid
RER: respiratory exchange ratio
RPE: ratings of perceived exertion
RPM: revolutions per min
TTR: tracer to tracee ratio
VCO2: volume of carbon dioxide expelled
VE: ventilatory exchange
VO2: volume of oxygen consumed
VO2: Max maximum oxygen uptake
WMax: maximal power.

## References

[1] Cahill GF. Starvation in man. N Engl J Med. (1970) 320:668–75. 10.1056/NEJM197003192821209 4915800

[2] Owen OE Morgan AP Kemp HG Sullivan Herrera MG Cahill GF. Brain metabolism during fasting. J Clin Investig. (1967) 46:1589–95. 10.1172/JCI105650 6061736 PMC292907

[3] Robinson AM Williamson DH. Physiological roles of ketone bodies as substrates and signals in mammalian tissues. Physiol Rev. (1980) 60:143–87. 10.1152/physrev.1980.60.1.143 6986618

[4] Balasse E Fery F. Ketone body production and disposal: effects of fasting, diabetes, and exercise. Diabetes Metab Rev. (1989) 5:247–70. 10.1002/dmr.5610050304 2656155

[5] Balasse EO Fery F Neef M. Changes induced by exercise in rates of turnover and oxidation of ketone bodies in fasting man. J Appl Physiol Respir Environ Exerc Physiol. (1978) 44:5–11. 10.1152/jappl.1978.44.1.5 627499

[6] Dearlove DJ Harrison OK Hodson L. The effect of blood ketone concentration and exercise intensity on exogenous ketone oxidation rates in athletes. Med Sci Sports Exerc. (2021) 53:505–16. 10.1249/MSS.0000000000002502 32868580 PMC7886359

[7] Kemp GJ Thompson CH Barnes PR. Comparisons of ATP turnover in human muscle during ischemic and aerobic exercise using 31P magnetic resonance spectroscopy. Magn Reson Med. (1994) 31:248–58. 10.1002/mrm.1910310303 8057795

[8] Margolis LM O'Fallon KS. Utility of ketone supplementation to enhance physical performance: a systematic review. Adv Nutr. (2020) 11:412–9. 10.1093/advances/nmz104 31586177 PMC7442417

[9] Evans M Cogan KE Egan B. Metabolism of ketone bodies during exercise and training: physiological basis for exogenous supplementation. J Physiol. (2017) 595:2857–71. 10.1113/JP273185 27861911 PMC5407977

[10] Wahren J Hagenfeldt L Felig P. Splanchnic and leg exchange of glucose, amino acids, and free fatty acids during exercise in diabetes mellitus. J Clin Investig. (1975) 55:1303–14. 10.1172/JCI108050 1133176 PMC301886

[11] Wahren J Sato Y Ostman J. Turnover and splanchnic metabolism of free fatty acids and ketones in insulin-dependent diabetics at rest and in response to exercise. J Clin Investig. (1984) 73:1362–76. 10.1172/JCI111340 6715541 PMC425159

[12] Randle PJ Garland PB Hales CN. The glucose fatty-acid cycle. Its role in insulin sensitivity and the metabolic disturbances of diabetes mellitus. Lancet. (1963) 1:785–9. 10.1016/S0140-6736(63)91500-9 13990765

[13] Hue L Taegtmeyer H. The Randle cycle revisited: a new head for an old hat. Am J Physiol Endocrinol Metab. (2009) 297:E578–91. 10.1152/ajpendo.00093.2009 19531645 PMC2739696

[14] Petrick HL Brunetta HS Pignanelli C. *In vitro* ketone-supported mitochondrial respiration is minimal when other substrates are readily available in cardiac and skeletal muscle. J Physiol. (2020) 598:4869–85. 10.1113/JP280032 32735362

[15] Taegtmeyer H. On the inability of ketone bodies to serve as the only energy providing substrate for rat heart at physiological work load. Basic Res Cardiol. (1983) 78:435–50. 10.1007/BF02070167 6626122

[16] Murray B Rosenbloom C. Fundamentals of glycogen metabolism for coaches and athletes. Nutr Rev. (2018) 76:243–59. 10.1093/nutrit/nuy001 29444266 PMC6019055

[17] Winwood-Smith HS Franklin CE White CR. Low-carbohydrate diet induces metabolic depression: a possible mechanism to conserve glycogen. Am J Physiol Regul Integr Comp Physiol. (2017) 313:R347–56. 10.1152/ajpregu.00067.2017 28701319

[18] Currell K Jeukendrup AE. Superior endurance performance with ingestion of multiple transportable carbohydrates. Med Sci Sports Exerc. (2008) 40:275–81. 10.1249/mss.0b013e31815adf19 18202575

[19] Holdsworth DA Cox PJ Kirk T. A ketone ester drink increases postexercise muscle glycogen synthesis in humans. Med Sci Sports Exerc. (2017) 49:1789–95. 10.1249/MSS.0000000000001292 28398950 PMC5556006

[20] Thomas DT Erdman KA Burke LM. Position of the academy of nutrition and dietetics, dietitians of Canada, and the American College of sports medicine: nutrition and athletic performance. J Acad Nutr Diet. (2016) 116:501–28. 10.1016/j.jand.2015.12.006 26920240

[21] Burke LM. Ketogenic low-CHO, high-fat diet: the future of elite endurance sport? J Physiol. (2021) 599:819–43. 10.1113/JP278928 32358802 PMC7891323

[22] Cox PJ Kirk T Ashmore T. Nutritional ketosis alters fuel preference and thereby endurance performance in athletes. Cell Metab. (2016) 24:256–68. 10.1016/j.cmet.2016.07.010 27475046

[23] Beylot M Beaufrère B Normand S. Determination of human ketone body kinetics using stable-isotope labelled tracers. Diabetologia. (1986) 29:90–6. 10.1007/BF00456116 3699302

[24] Chong MFF Fielding BA Frayn KN. Mechanisms for the acute effect of fructose on postprandial lipemia. Am J Clin Nutr. (2007) 85:1511–20. 10.1093/ajcn/85.6.1511 17556686

[25] van Loon LJ Saris WH Krushoop M. Maximizing postexercise muscle glycogen synthesis: Carbohydrate supplementation and the application of amino acid or protein hydrolysate mixtures. Am J Clin Nutr. (2000) 72:106–11. 10.1093/ajcn/72.1.106 10871568

[26] Faul F Erdfelder E Lang A-G. G^*^Power 3: a flexible statistical power analysis program for the social, behavioral, and biomedical sciences. Behav Res Methods. (2007) 39:175–91. 10.3758/BF03193146 17695343

[27] Mikkelsen KH Seifert T Secher NH. Systemic, cerebral and skeletal muscle ketone body and energy metabolism during acute hyper-D-V-hydroxybutyratemia in post-absorptive healthy males. J Clin Endocrinol Metab. (2015) 100:636–43. 10.1210/jc.2014-2608 25415176

[28] van Loon LJ Greenhaff PL Constantin-Teodosiu D. The effects of increasing exercise intensity on muscle fuel utilisation in humans. J Physiol. (2001) 536:295–304. 10.1111/j.1469-7793.2001.00295.x 11579177 PMC2278845

[29] Johnson RH Walton JL. The effect of exercise upon acetoacetate metabolism in athletes and non-athletes. Q J Exp Physiol Cognate Med Sci. (1972) 57:73–9. 10.1113/expphysiol.1972.sp002139 4482033

[30] Hearris MA Hammond KM Fell JM. Regulation of muscle glycogen metabolism during exercise: implications for endurance performance and training adaptations. Nutrients. (2018) 10:298. 10.3390/nu10030298 29498691 PMC5872716

