# Supplementary material for: β-Hydroxybutyrate Oxidation in Exercise Is Impaired by Low-Carbohydrate and High-Fat Availability
Source: Front Med (Lausanne). 2021 Nov 25;8:721673. doi: 10.3389/fmed.2021.721673 (PMC8655871; doi:10.3389/fmed.2021.721673)
Supplement: Supplementary file 1 [file Data_Sheet_1.PDF]

SUPPLEMENTARY INFORMATION

RUNNING TITLE: KETONE OXIDATION AND SUBSTRATE AVAILABILITY

Authors:

DAVID J. DEARLOVE[†*] DAVID HOLDSWORTH[†*], TOM KIRK[†], LEANNE HODSON[‡], EVELINA CHARIDEMOU[§], ELINE KVALHEIM[#], BRIANNA STUBBS[†], ANDREW BEEVERS[¶], JULIAN L. GRIFFIN[§], RHYS EVANS[†], JEREMY ROBERTSON[#], KIERAN CLARKE[†], PETE J. COX[†]

[*]Co-first author
[†]Department of Physiology, Anatomy and Genetics, University of Oxford
[‡]Oxford Centre for Diabetes, Endocrinology and Metabolism, and Oxford NIHR Biomedical Research Centre, University of Oxford
[§] Department of Biochemistry and Cambridge Systems Biology Centre, University of Cambridge and MRC Human Nutrition Research, Cambridge, UK
[#]Department of Chemistry, University of Oxford UK.
[¶] Department of Chemistry, Sterling Pharmaceutical Solutions Ltd, UK

Corresponding author: DAVID J. DEARLOVE
Department of Physiology, Anatomy and Genetics
The University of Oxford
david.dearlove@dpag.ox.ac.uk
+44 7739460623

Submitted: Thursday 7th October, 2021

Nonstandard abbreviations

**βHB**	β-hydroxybutyrate
**W_{Max}**	maximal power

Supplementary Information

S1. Example daily diet diary.

Table S1

Time	Item	A/V	Fat (g)	CHO (g)	Protein (g)	Kcals (total)
21:00	Oats	100 g	8	60	11	150
	Spirulina powder	5 g	0	1	3	18
	Honey	25 g	0	21	0	76
	Sunflower seeds	12 g	6	2	3	70
	Semi-skimmed milk	150 ml	2	5	4	54
	Banana malt loaf	260 g	13	145	19	793
5:50	Granola	240 g	30	151	26	1015
	Oats	50 g	4	30	6	75
	Sprillina	5 g	0	1	3	18
	Sunflower seeds	12 g	6	2	3	70
	Honey	10 g	0	8	0	30
	Prunes	12 g	0	4	0	19
	Semi-skimmed milk	200 ml	3	7	5	72
8:30	Oats	100 g	8	60	11	150
	Banana	1 med	0	23	1	89
	Goji berries	10 g	0	6	1	31
	Semi-skimmed milk	150 ml	2	5	4	54
12:00	Sour dough bread	54 g	0	22	3	102
	Avocado	90 g	14	8	2	144
	Eggs	2	10	1	12	156
	Low fat Greek yogurt	300 g	28	14	13	369
16:00	Granola	250 g	32	157	27	1058
19:10	Couscous	150 g	0	35	6	168
	Roasted vegetable mix	240 g	2	11	2	84
	Mint chocolate	88 g	28	46	5	473
			**197**	**827**	**169**	**5337**

S2. Skeletal muscle metabolites

Metabolite extractions: Metabolites were extracted using the Folch method [1]. In Brief, ≈20 mg of muscle tissue was homogenised in chloroform/methanol (2:1; 750 μL) using a TissueLyser (Qiagen, Germany). After complete homoginisation, water (300 μL) was added and the samples were mixed thoroughly. The layers were separated by centrifuging at 13,000 g for 20 min. The aqueous fraction (upper layer) was collected and dried in a CentriVap Centrifugal Concentrator with attached cold trap (78100 series, Labconco, US). The organic fraction (lower fraction) was dried under a stream of nitrogen.

Liquid chromatography-mass spectrometry: The aqueous fraction was reconstituted in 100 μL of a acetonitrile:10 mM ammonium carbonate water solution (7:3 v/v) containing a mixture of eight internal standards at the concentration of 10 μM (AMP $^{13}C_{10}$, $^{15}N_5$, ATP $^{13}C_{10}$, $^{15}N_5$ Glutamate U^{13}C, U^{15}N Leucine d_{10}, Phenylalanine d_5, Proline U^{13}C, U^{15}N, Serotonin d_4 and Valine d_8). Samples were injected onto a Thermo Scientific UHPLC+ series coupled with a TSQ Quantiva mass spectrometer (Thermo Fisher Scientific, USA) with an ESI source, operated in positive and negative ion mode at the same time. The electrospray voltage was set to 3500 V for the positive ionisation and to 2500 V for the negative ionisation.

Normal phase analysis: The samples were analysed with a BEHAmide (150 x 2.1 mm 1.7 μm) column. The column was conditioned at 30 °C. The mobile phase consisted of: (A) a 0.1% of ammonium carbonate water solution and (B) an acetonitrile solution. The mobile phase was pumped at a flow rate of 600 μL·min^{-1} programmed as follows: started at 80% of B for 1.5 min followed by a linear decrease from 80% to 40% of B for 3.5 min and finally, returned to initial conditions.

Reverse phase analysis: Samples were dried and reconstituted 10 mM ammonium acetate water solution and analysed with an ACE Excel 2 C18 PFP (100A. 150 x 2.1 mm 5 μ) column. The column was conditioned at 30 °C. The mobile phase consisted of: (A) a 0.1% of formic acid water solution and (B) a 0.1% of formic acid in acetonitrile solution. The mobile phase was pumped at a flow rate of 500 μL·min^{-1} programmed as follows: started at 0% of B for 1.6 min followed by a linear increase from 0% to 30% of B at 4 min and to 90% at 4.5 min for 1 min and then returned back to initial conditions.

Carnitine analysis: The aqueous and organic fractions were combined in equal parts, dried and then reconstituted in 4:1 methanol:water containing carnitine IS mix. Samples were injected onto an ACE Excel 2 C18 PFP (100A. 150 x 2.1 mm 5 μ) column. The column was conditioned at 30 °C. The mobile phase consisted of: (A) a 0.1% of formic acid water solution and (B) a 0.1% of formic acid in methanol solution. The mobile phase was pumped at a flow rate of 500 μL·min^{-1} programmed as follows: started at 0.5% B for 1 min, then gradually increased to 100% B at 9 min for 2 min and returned back to initial conditions.

Metabolite analysis: The Xcalibur software (Thermos fisher scientific, Waltham, Massachusetts, USA) was used for data acquisition. Putative recognition of all detected metabolites was performed using a targeted MS/MS analysis.

S3. Pre-visit dietary composition

Data (mean ± SEM) are taken from participant reported diet diaries and represent total food intake for the period between the glycogen depleting exercise and evening prior to study visits (≈36 hours).

Table S2

	KE+CHO	KE+CHO+FAT	KE+FAT
Carbohydrate	63.1 ± 1.7%	65.7 ± 4.7%	3.3 ± 0.4%
Fat	26.5 ± 3%	23.5 ± 6%	69.4 ± 1.2%
Protein	10.4 ± 1.7%	10.8 ± 2.4%	27.3 ± 1.4%
Total energy (Kcal)	5539 ± 496	5871 ± 751	5580 ± 559

S4. β-hydroxybutyrate (βHB) oxidation rates at rest (0 min) and during exercise at 70% maximal power (W_{Max}) (25 min, 45 min and 60 min).

Resting βHB oxidation rates were analysed by 1-way repeated measures ANOVA (ANOVA effect, p=0.3) with exercise βHB oxidation rates analysed separately within a 2-way repeated measures ANOVA (Condition, p=0.2; Condition*Time interaction, p=0.6). Given the lack of ANOVA effects, all post-hoc comparisons are exploratory.

Table S3

	βHB ox. rate mean diff. (g·min^{-1})	95% CI	Cohen's *d*	*p*
Rest (0 min)				
KE+CHO vs. KE+CHO+FAT	0.01	-0.01 to 0.03	0.6	0.5
KE+CHO vs. KE+FAT	0.02	-0.01 to 0.04	0.9	0.2
KE+CHO+FAT vs. KE+FAT	0.01	-0.02 to 0.03	0.5	0.9
Exercise (25 min)				
KE+CHO vs. KE+CHO+FAT	<0.01	-0.02 to 0.02	<0.1	>0.9
KE+CHO vs. KE+FAT	0.03	0.01 to 0.05	0.7	0.001
KE+CHO+FAT vs. KE+FAT	0.03	0.01 to 0.05	0.5	0.001
Exercise (45 min)				
KE+CHO vs. KE+CHO+FAT	-0.01	-0.03 to 0.01	0.2	0.3
KE+CHO vs. KE+FAT	0.03	0.02 to 0.05	0.7	<0.001
KE+CHO+FAT vs. KE+FAT	0.05	0.03 to 0.06	0.6	<0.001
Exercise (60 min)				
KE+CHO vs. KE+CHO+FAT	<0.01	-0.28 to 0.01	0.2	0.5
KE+CHO vs. KE+FAT	0.03	0.01 to 0.05	0.6	<0.001
KE+CHO+FAT vs. KE+FAT	0.04	0.02 to 0.06	0.6	<0.001

S5. Intramuscular carnitine species.

FIGURE S1 ABOUT HERE.

A) Free carnitine

B) C2 acylcarnitine

C) C4 acylcarnitine

D) C12 acylcarnitine
E) C14:1 acylcarnitine
F) C14 acylcarnitine
G) C16:1 acylcarnitine
H) C16 acylcarnitine
I) C18:1 acylcarnitine
J) C18:2 acylcarnitine
K) C18 acylcarnitine
Significant ANOVA effects are presented in relevant figures. Significant post-hoc comparisons: † = KE+CHO vs. KE+FAT; ‡ = KE+CHO+FAT vs. KE+FAT; § = KE+CHO vs. KE+CHO+FAT; ¶ = Pre- vs. post-exercise. Values = mean ± SD.

References

[1] J. Folch, M. Lees, and G. H. Sloane Stanley. “A simple method for the isolation and purification of total lipides from animal tissues.” In: *The Journal of biological chemistry* 226.1 (1957), pp. 497–509.
