# Supplementary figures and images for: β-Hydroxybutyrate Oxidation in Exercise Is Impaired by Low-Carbohydrate and High-Fat Availability
Source: Front Med (Lausanne). 2021 Nov 25;8:721673. doi: 10.3389/fmed.2021.721673 (PMC8655871; doi:10.3389/fmed.2021.721673)

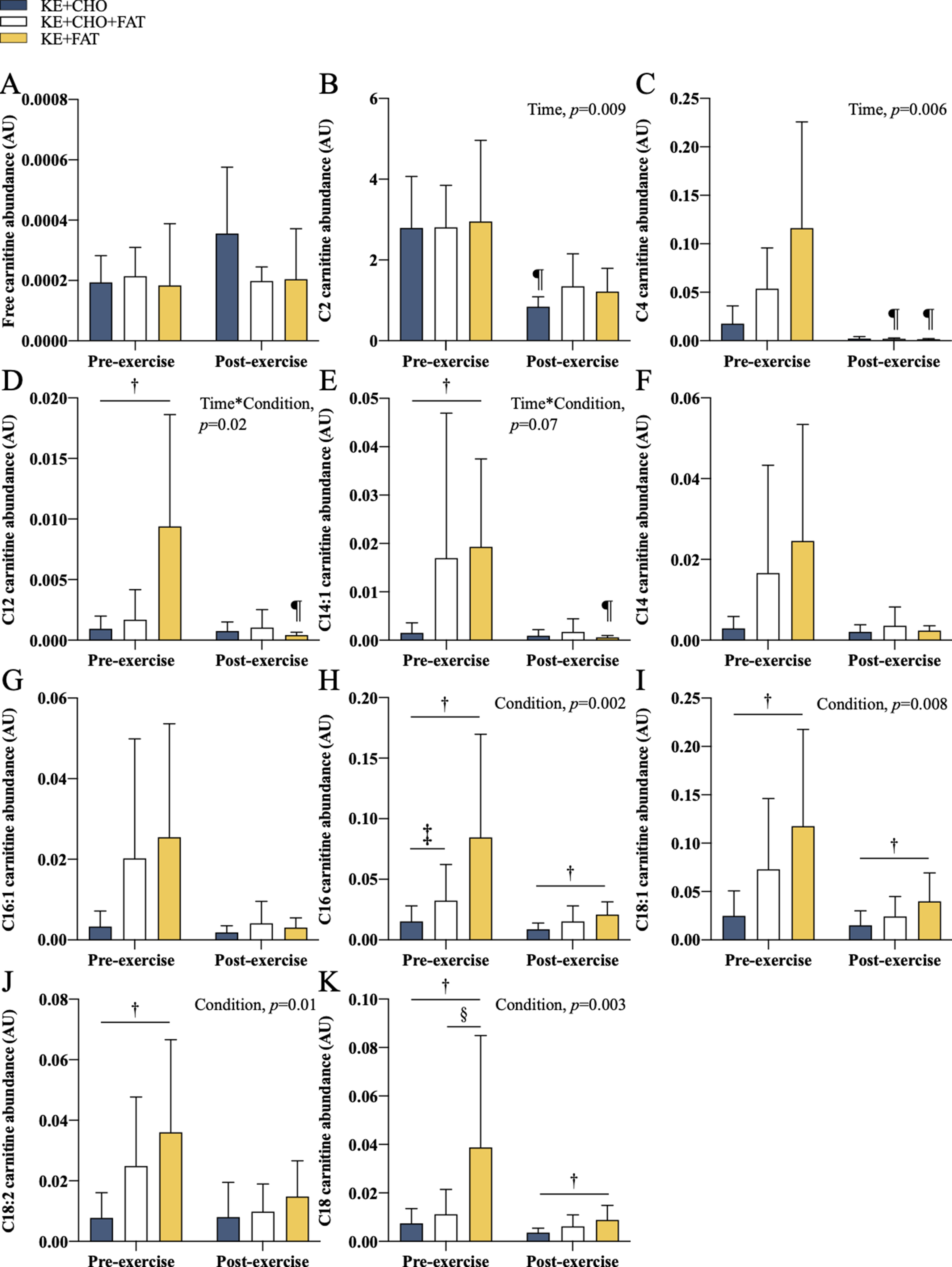

Supplement: Supplementary file 2 [file Image_1.TIFF]
